# A Laboratory Study of Listerine

**Published:** 1882-09-20

**Authors:** Frank M. Deems

**Affiliations:** President Augusta (Ga.) Academy of Medicine; formerly Laboratory Instructor in the Medical Department of the University of New York; Member of the New York Microscopical Society


### A LABORATORY STUDY OF LISTERINE.

By FRANK M. DEEMS, M. D., PH. D.,

President Augusta (Ga.) Academy of Medicine; formerly Laboratory Instructor in the Medical Department of the University of New York; Member of the New York Microscopical Society.

Herewith I submit the report of the investigation I have made with the antiseptic combination known as Listerine. The preparation itself is a clear liquid, of an acid reaction, a powerful, fragrant, aromatic odor and pungent taste, both of which are rather pleasant and agreeable than otherwise. Its specific gravity is considerably lighter than that of water, with which, however, it is readily miscible in any proportion.

Listerine is anti-zymotic in the strict sense of the word, as derived from the Greek *anti*. against, and *zumosis*, fermentation.

Without entering here into a discussion of the question as to whether or not fermentation of every sort (be it alcoholic, acetic, lactic, mannitic, butyric, ammoniacal, or putrefactive) is due to the action, and formed under the influence of, living organisms on the material undergoing change, "it will be admitted on all sides, first, that these living entities are the invariable accompaniments, under ordinary circumstances, of fermentative processes; second, that substances which poison or kill these germs likewise avert these processes."* Anti-zymotics, therefore, are substances used for the purpose of preventing decomposition, but their most important use is to kill disease-germs—to destroy the activity of the living particles which constitute contagion. In this sense I believe Listerine is, from numerous, varied and repeated tests—the details of which I append to this report—a powerful and trustworthy antiseptic agent. It prevents the various fermentations.

Meat keeps indefinitely in Listerine. It is a swift and sure destroyer of infusorial life. It destroys the activity, growth and motion of low forms of vegetable life. Owing to this property, combined with its non-toxic effect on the human system in quantities medicinal and not excessive, it has the great advantage over carbolic acid in that it may be administered *internally* as well as used with freedom either by injection, lotion, or spray in the natural cavities of the body, such as the ears, nose, mouth, throat, larynx, trachea, bronchial tubes, rectum, vagina, urethra and bladder. Even in full strength Listerine does not coagulate the albumen of the flesh. I believe that, owing to its germ-destroying power and non-poisonous action, it is peculiarly adapted to the treatment of diseases affecting these parts, especially to those calling for an antiseptic remedy. Inasmuch as there is a great difference between the environment of germs in ordinary fermentations outside of the body (as in the experiments below recorded) and those in the organism, it is evident that doses and *dilutions* of antiseptics generally, and of Listerine in particular, harmless to the former, may have very great effect against the latter, because in the artificially-prepared fluids of the laboratory the micro-organisms only find comparatively inert matter, whereas in the organism they have to contend against the vitality of the globules, "which are in themselves a sort of living beings."

I have endeavored, as far as possible, to indicate the dilutions required in practice, but this point can best be settled by experience. Keeping the above statement in view, however, I believe the experiments warrant somewhat greater dilutions than those recorded in the experiments and conclusions. Pending my investigations of its power over ferments, I have used it in my daily practice, and so far my clinical experience has confirmed my expectations of its efficacy. It is an agreeable and perfect tooth and mouth wash. I have used it with success in purulent conjunctivitis (diluted one-third), and two cases of leucorrhea yielded promptly to its use. I shall look for excellent results from its administration during the summer in the various diarrheal diseases of that season, and especially in those affecting children.

### CONCLUSIONS.†

Listerine up to a dilution of ten per cent. prevents putrefaction and preserves animal tissues. This solution being "sterilized" is analogous to the conditions of a freshly-made wound, and indicates

†We have carefully examined the tables reporting the conditions of Dr. Deems's experiments, and they fully justify these conclusions. We regret we have not space for the tables, which are very complete and elaborate.—*Eds. Louisville Med. News.*

*Prof. H. C. Wood.

the safety of Listerine when employed to prevent the introduction and growth of germs.

Animal tissues are preserved in it (full strength), and no putrefaction can occur in tissues immersed in it.

A twenty-five-per-cent solution of it prevents the development of bacteria and fungi in urine, and a five-per-cent. solution retards the usual changes which this excretion undergoes. This seems to recommend for cystitis and other vesical diseases a dilution of from one to four to one to ten parts as an injection into the bladder.

Fresh milk mixed with it, in the proportion of one of the latter to ten of the former, will keep wholesome for a week during warm weather. One to twenty will retard the changes sufficiently to make it a desirable article in the sick-room. This indicates an important use in typhoid fever and intestinal troubles generally.

A thirty-three-and-a-third-per-cent. solution of it prevents the development of bacteria, and consequently the decomposition of a vegetable infusion.

A fifty-per-cent. solution of it arrests the development of bacteria in a vegetable infusion.

